# Materials science optimization benchmark dataset for multi-objective, multi-fidelity optimization of hard-sphere packing simulations

**DOI:** 10.1016/j.dib.2023.109487

**Published:** 2023-08-10

**Authors:** Sterling G. Baird, Ramsey Issa, Taylor D. Sparks

**Affiliations:** a Materials Science & Engineering, 122 S. Central Campus Drive, #304 Salt Lake City, UT 84112-0056, United States; b Chemistry Department, University of Liverpool, Liverpool, L7 3NY, United Kingdom

**Keywords:** Adaptive design, Physics-based, Lubachevsky–Stillinger, Force-biased algorithms, Particle packing, Packing generation, Transfer learning, Size distribution

## Abstract

In scientific disciplines, benchmarks play a vital role in driving progress forward. For a benchmark to be effective, it must closely resemble real-world tasks. If the level of difficulty or relevance is inadequate, it can impede progress in the field. Moreover, benchmarks should have low computational overhead to ensure accessibility and repeatability. The objective is to achieve a kind of ``Turing test'' by creating a surrogate model that is practically indistinguishable from the ground truth observation, at least within the dataset's explored boundaries. This objective necessitates a large quantity of data. This data encompasses numerous features that are characteristic of chemistry and materials science optimization tasks that are relevant to industry. These features include high levels of noise, multiple fidelities, multiple objectives, linear constraints, non-linear correlations, and failure regions. We performed 494498 random hard-sphere packing simulations representing 206 CPU days’ worth of computational overhead. Simulations required nine input parameters with linear constraints and two discrete fidelities each with continuous fidelity parameters. The data was logged in a free-tier shared MongoDB Atlas database, producing two core tabular datasets: a failure probability dataset and a regression dataset. The failure probability dataset maps unique input parameter sets to the estimated probabilities that the simulation will fail. The regression dataset maps input parameter sets (including repeats) to particle packing fractions and computational runtimes for each of the two steps. These two datasets were used to create a surrogate model as close as possible to running the actual simulations by incorporating simulation failure and heteroskedastic noise. In the regression dataset, percentile ranks were calculated for each group of identical parameter sets to account for heteroskedastic noise, thereby ensuring reliable and accurate data. This differs from the conventional approach that imposes a-priori assumptions, such as Gaussian noise, by specifying mean and standard deviation. This technique can be extended to other benchmark datasets to bridge the gap between optimization benchmarks with low computational overhead and the complex optimization scenarios encountered in the real world.

Specifications Table

**Table:** 

Subject	Computational materials science
Specific subject area	Physics-based geometric packing
Type of data	Table Figure
How the data were acquired	Data was acquired by running compiled C software hosted at https://github.com/VasiliBaranov/packing-generation in a two-step process orchestrated using Python in https://github.com/sparks-baird/matsci-opt-benchmarks/blob/main/scripts/particle_packing/packing_generation_submitit.py. The Python code was utilized as a driver for the compiled packing generation executable and executed using the resources provided by the University of Utah's Center for High-performance Computing (CHPC). The submission of jobs to the SLURM scheduler was facilitated through https://github.com/facebookincubator/submitit, and the MongoDB Data API was utilized to record data in JSON format. For a snapshot of the code utilized in matsci-opt-benchmarks, please refer to https://github.com/sparks-baird/matsci-opt-benchmarks/tree/v0.2.2 (https://zenodo.org/record/7697264#.ZAJo6nbMIeM).
Data format	Raw Analyzed Filtered
Description of data collection	We use geometry-based particle packing simulations to generate a set of spheres within a volume and analyze the packing fraction (occupied space vs. total space). A total of 65536 parameter combinations were randomly sampled using quasi-random Sobol sampling, varying seven irreducible parameters in addition to the number of particles and initial scaling factor. A constrained search space was employed through the Ax Platform with repeats. Out of these simulations, 494498 were successfully completed, requiring 206 CPU days to run. Packing simulations were run using two algorithms run sequentially (i.e., a two-step process). Sometimes, the algorithms can fail. For example, during an approximate search of neighboring particles, sometimes not all neighboring particles are found. Failed simulations were recorded as NaN values with ratio
	of successful to total simulations tracked on a per parameter set basis (sobol_probability_filter.csv). Repeat simulations were grouped and ranked by percentile using the “dense” method with pct=True in pandas.core.groupby.GroupBy.rank (sobol_regression.csv) [1]. Surrogate models were fitted for failure probability, packing fraction, and computational runtime for each of two particle packing algorithms, totaling six surrogate models.
Data source location	University of Utah, Salt Lake City UT USA
Data accessibility	Repository name: Zenodo Data identification number: 7696165 Direct URL to data: https://dx.doi.org/10.5281/zenodo.7696165

## 1 Value of the Data

• Valuable for adaptive design benchmarking
• Benefits optimization researchers and practitioners in the physical sciences
• Provides insight into packing behavior in powder-bed additive manufacturing, can be integrated with experimental data
• Provides an example for future datasets

## 2 Objective

Optimization tasks that are relevant to industry in the fields of materials science and chemistry are typically hierarchical, noisy, multi-fidelity [2,3], multi-objective [4,5], high-dimensional [6,7], non-linearly correlated, and involve mixed numerical and categorical variables subject to linear [8] and non-linear constraints. Existing benchmark datasets [9], [10], [11], [12], [13], [14] have limitations as they ignore or simplify the impact of noise and the occurrence of failure with certain parameter combinations. By integrating simulation failure and heteroskedastic noise, we aim to achieve a ``Turing test'' scenario where the surrogate model is practically indistinguishable from the ground truth simulation. This strategy bridges the gap between low-cost surrogate functions based on benchmark datasets and the high-cost evaluation of objective functions in real-world scenarios.

## 3 Data Description

The failure probability dataset (sobol_probability_filter.csv) contains unique input parameter sets (nine variables) and the estimated probabilities that the simulation will fail at each of the two steps (force-biased algorithm [15,16] and Lubachevsky–Stillinger [17], [18], [19]).

The regression dataset (sobol_regression.csv) contains input parameters (including repeats) spanning nine variables and corresponding particle packing fractions as well as computational runtimes for each of the two steps (force-biased algorithm and Lubachevsky–Stillinger).

There are six regression models (surrogate_models.pkl) trained on all data meant for production use. These six models can be used together to create the benchmark function.

There are five cross-validation sets of six regression models (cross_validation_models_0.pkl, cross_validation_models_1.pkl, cross_validation_models_2.pkl, cross_validation_models_3.pkl, cross_validation_models_4.pkl).

The model metadata (model_metadata.json) contains the raw mean absolute error scores, the raw predictions, and the true values for each of the cross-validation folds.

**Fig. 1:**
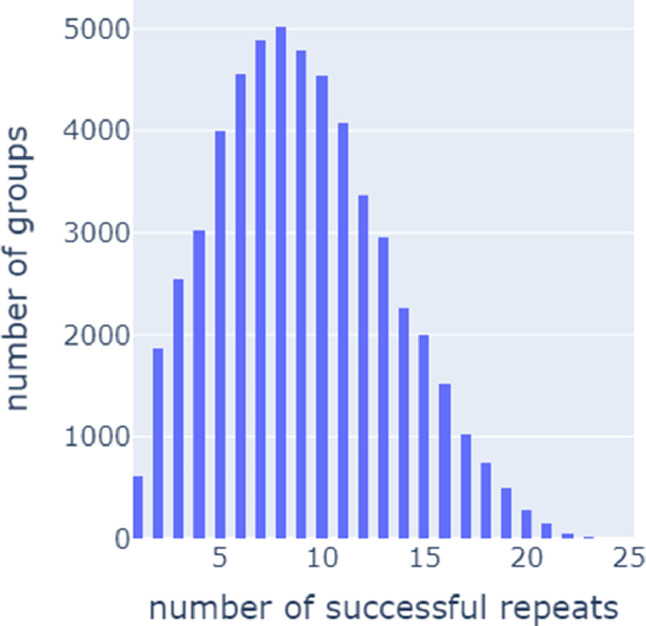
Histogram of number of parameter groups vs. number of successful repeats within a given group. For example, of the 65536 unique parameter combinations, approximately 5000 had eight successful repeats.

For each group of repeats, we tracked the number of simulations that were run and the number of simulations that ran successfully. Fig. 1 contains a histogram of the number of successful repeats for each parameter combination. For example, of the 65536 unique parameter combinations, approximately 5000 had eight successful repeats.

**Fig. 2:**
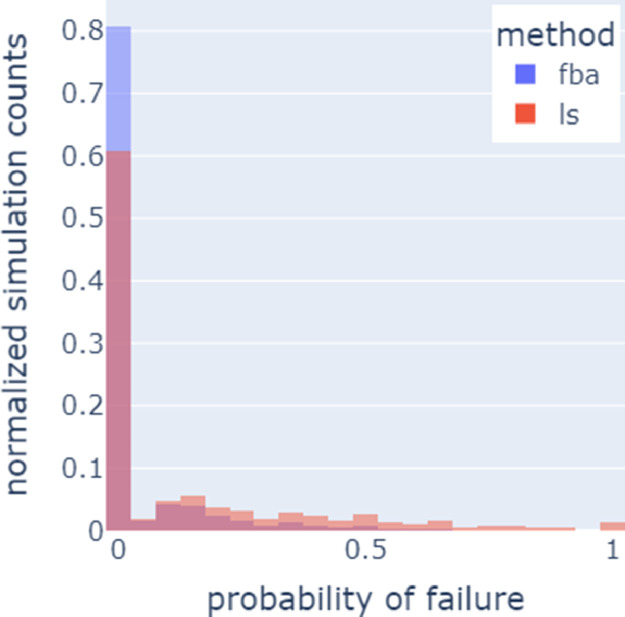
Histogram of normalized simulation counts vs. the probability of a simulation failing for a given parameter set. On average, the force-biased algorithm or fba (blue) is more likely to succeed than the Lubachevsky–Stillinger or ls (red) algorithm.

For a given parameter set, the probability of a simulation failing is the number of failed simulations divided by the number of simulations that were run. Fig. 2 contains the probabilities of a parameter set failing for each of the two algorithms (force-biased and Lubachevsky–Stillinger).

**Fig. 3:**
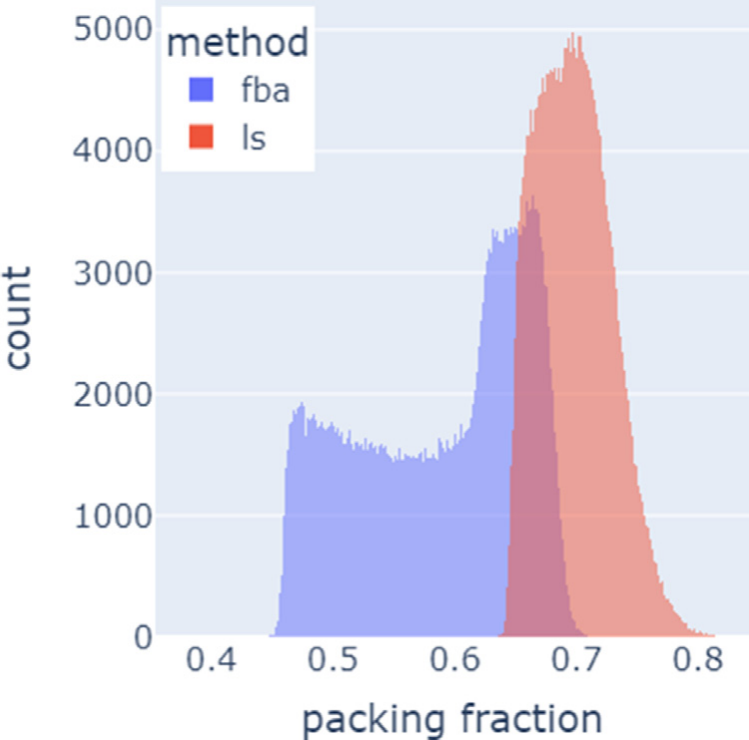
Histogram of number of simulations vs. packing fraction for the force-biased algorithm or fba (blue) and Lubachevsky–Stillinger or ls algorithm (red). On average, the ls algorithm tends to have higher packing fractions with a more Gaussian-like distribution than fba.

Fig. 3 contains the histograms of observed particle packing fractions for each of the two algorithms.

## 4 Experimental Design, Materials and Methods

For this dataset, we aim to achieve a “Turing test” scenario where the surrogate model for a simulation is practically indistinguishable from the corresponding ground truth simulation. Here, we use https://github.com/VasiliBaranov/packing-generation [20] to run hard-sphere particle packing simulations while varying the particle size distribution. We ran repeat simulations to better capture noise, and we also tracked when simulations fail and the computational runtime at each step. Particle packing simulations were performed in a two-step process of a force-biased algorithm [15,16] followed by the Lubachevsky–Stillinger algorithm [17], [18], [19]. An attempt to run the LS algorithm was always preceded by an attempt to run the FBA algorithm. If the force-biased algorithm failed, the Lubachevsky–Stillinger algorithm was still attempted (https://github.com/sparks-baird/matsci-opt-benchmarks/blob/v0.2.2/src/matsci_opt_benchmarks/particle_packing/utils/packing_generation.py#L63-L183). The simulations were performed using mixtures of three different particle types, each characterized by two log-normal distribution parameters and three composition parameters. Two parameters (scale and shape) describe each of the three distributions, and three additional composition parameters describe the fractional share (e.g., in terms of volume) of each of the particle types. These nine parameters fully define the particle size distribution. With appropriate constraints applied, only seven of these parameters are necessary to fully define the particle size distribution. Additionally, the number of particles and an initial scaling factor were allowed to vary. With a greater number of particles, denser and more realistic packs can be generated at the expense of computational cost (i.e., the fidelity parameter). The initial scaling factor affects the computational stability of the simulation; with an adequate scaling factor, the simulation is more likely to be completed successfully. The quasi-random Sobol sampling technique was employed to generate parameter combinations, enabling a more uniform sampling of the allowable parameter space. We sampled 65536 unique parameter combinations. Repeat simulations for the parameter combinations were run to capture heteroskedastic noise, totaling 494498 simulations. To increase throughput and reduce latency, simulation parameters (including repeats) were shuffled and divided into batches, which were then dispatched to a high-performance computing environment for asynchronous evaluation. The data were recorded in a free-tier MongoDB Atlas database and then consolidated and prepared as datasets suitable for machine learning applications. Although it may serve other purposes, this dataset was primarily designed as a multi-fidelity benchmark dataset for constrained adaptive design experiments, hence the tracking of repeats, running simulations at various fidelities, incorporation of constraints, and tracking when simulations fail and the computational expense (whether or not the simulation runs successfully). For further implementation details, see https://github.com/sparks-baird/matsci-opt-benchmarks/tree/v0.2.2/scripts/particle_packing and https://github.com/sparks-baird/matsci-opt-benchmarks/tree/v0.2.2/notebooks/particle_packing. Instructions for model usage are available at https://matsci-opt-benchmarks.readthedocs.io/.

## Ethics Statement

There are no statements to declare.

## CRediT authorship contribution statement

**Sterling G. Baird:** Project administration, Conceptualization, Methodology, Software, Validation, Formal analysis, Investigation, Data curation, Writing – original draft, Writing – review & editing, Visualization. **Ramsey Issa:** Methodology, Software, Validation, Formal analysis, Writing – review & editing. **Taylor D. Sparks:** Supervision, Funding acquisition.

## Data Availability

Materials Science Optimization Benchmark Dataset for Multi-Objective, Multi-Fidelity Optimization of Hard-Sphere Packing Simulations (Original data) (Zenodo)
